# Breeding has selected for architectural and photosynthetic traits in lentils

**DOI:** 10.3389/fpls.2022.925987

**Published:** 2022-08-25

**Authors:** Viridiana Silva-Perez, Arun S. K. Shunmugam, Shiwangni Rao, C. Mariano Cossani, Abeya Temesgen Tefera, Glenn J. Fitzgerald, Roger Armstrong, Garry M. Rosewarne

**Affiliations:** ^1^Agriculture Victoria, Horsham, VIC, Australia; ^2^School of Agriculture, Food and Wine, South Australian Research and Development Institute, The University of Adelaide, Urrbrae, SA, Australia; ^3^Centre for Agricultural Innovation, School of Agriculture and Food, Faculty of Veterinary and Agricultural Sciences, The University of Melbourne, Parkville, VIC, Australia; ^4^Department of Animal, Plant and Soil Sciences, La Trobe University, Melbourne, VIC, Australia

**Keywords:** *Lens culinaris* Medik, CO_2_ assimilation rate, stomatal conductance, canopy temperature, RUE, SPAD, photosynthesis, LMA

## Abstract

Genetic progress in seed yield in lentils (*Lens culinaris* Medik) has increased by 1.1% per year in Australia over the past 27 years. Knowing which plant traits have changed through breeding during this time can give important insights as to how lentil yield has increased. This study aims to identify morphological and physiological traits that were directly or indirectly selected between 1993 and 2020 in the Australian lentil breeding program using 2 years of experimental data. Major changes occurred in plant architecture during this period. Divergent selection has seen the release of varieties that have sprawling to very upright types of canopies. Despite this genetic diversity in recently released varieties, there is an overall tendency of recently released varieties having increased plant height and leaf size with reduced number of branches. Increased light interception was positively correlated with year of release (YOR) and yield, and likely results from indirect selection of yield and taller plant types. There is an indication that recently released varieties have lower CO_2_ assimilation rate, stomatal conductance and canopy temperature depression (CTD) at high ambient temperatures (~30°C). Understanding lentil physiology will assist in identifying traits to increase yield in a changing climate with extreme weather events.

## Introduction

Pulses are an important source of protein in the human diet, with lentil protein being particularly rich in amino acids and bioactive peptides, which confer several health benefits (Khazaei et al., 2019). Australia’s lentil industry is relatively new but has increased production rapidly, from 183 t in 1991 to 525,848 t in 2020 (ABARES, 2021). Lentils, like other legumes, also provide environmental benefits by fixing nitrogen (N) in the soil, reducing the need for N fertilizers. When rotated with cereals, lentils help to break disease cycles, provide alternative herbicide options for weed control and leave residual N and water for subsequent crops (Evans et al., 2001; Kirkegaard et al., 2008; Angus et al., 2015).

The first lentil cultivars grown in Australia were introduced from The International Center for Agriculture Research in the Dry Areas (ICARDA) and Canadian germplasm. Adaptation to Australian conditions was tested in different regions of the country from 1988 to 1992, and the expansion of lentils in Victoria and South Australia started in 1993 (Materne and Brouwer, 1997). Lentil breeding programs have focused on yield, herbicide tolerance, marketability, phenology, disease resistance, plant height, and boron and salt tolerance (Materne and Brouwer, 1997; Brennan et al., 2002; Hobson et al., 2006).

Plant breeding has modified several traits through indirect selection for yield such as plant architecture (Richards et al., 2019) and radiation use efficiency (RUE; Sadras et al., 2012). RUE is defined as the conversion of radiation intercepted by green tissue into biomass and is expressed as grams of dry matter per megajoule of intercepted photosynthetically active radiation (Monteith, 1977). Compared with chickpea, lupin and field peas, lentils have low RUE and it has been suggested that there is scope to improve RUE to increase the yield of pulses (Ayaz et al., 2004). In Australian lentil germplasm, harvest index (HI) has been correlated with yield across multiple environments (Lake and Sadras, 2021) and the Australian lentil breeding program has also bred for plant architecture, particularly for dry environments where upright architecture facilitates mechanical harvest.

In wheat, light interception (LI), RUE and HI have been associated with improved yield (Reynolds et al., 2009). Variation in lentil canopy architecture has been moderately associated with genotypic variation in light interception (McKenzie and Hill, 1991; Hanlan et al., 2006). Plant height in wheat and leaf type in chickpeas have also been associated with changes in LI or RUE (Miralles and Slafer, 1997; Li et al., 2008). Studies in lentils have shown variation in architectural traits such as branch angle, number of branches and plant height (Erskine and Goodrich, 1991; Hanlan et al., 2006).

Photosynthesis has become a target for improving yield potential in wheat (Parry et al., 2011). A positive correlation between CO_2_ assimilation rate (*A*) with yield and year of release has been found, suggesting indirect selection for photosynthetic traits (Reynolds et al., 1994, 2000a; Fischer et al., 1998; Tang et al., 2017). This is seen as an untapped opportunity to improve yield, particularly as yield improvements through improved HI in cereals may have reached a plateau (Furbank et al., 2020). Soybean yield has also been shown to be correlated with higher photosynthesis (Buttery and Buzzell, 1972; Ojima, 1974; Morrison et al., 1999, 2000; Jin et al., 2010; Liu et al., 2012). In soybean, the more recently released cultivars have a higher daily carbon gain associated with a higher photosynthetic rate, concomitant with higher yields and HI compared with early released cultivars (Koester et al., 2016). Lentils grown under elevated CO_2_ have increased biomass and yield, and it has been suggested that studies assessing genetic variation in CO_2_ assimilation rates would be useful in understanding this response (Bourgault et al., 2017).

Genetic diversity in several leaf photosynthetic traits has been recorded in wheat, (Driever et al., 2014; Jahan et al., 2014; Carmo-Silva et al., 2017; Silva-Perez et al., 2020), rice (Acevedo-Siaca et al., 2020, 2021) and soybeans (Koester et al., 2016). To our knowledge, no such studies of CO_2_ assimilation rate and related traits have been published in lentils, let alone information on the extent of genotypic variation for these traits.

The aim of this study was to identify physiological and morphological traits in lentils that may have been indirectly selected by breeding from 1993 to 2020 in Australia in relation with yield, HI and biomass, and to detect the traits that can be incorporated in the breeding program. In this study, we analysed a wide range of physiological and morphological traits of lentil varieties released by the Australian breeding program and assess their contribution to improved yields over the last 27 years.

## Materials and methods

### Plant material and experimental design

Lentil germplasm for this study was obtained from ICARDA, Canada and subsequent releases by Agriculture Victoria’s Australian national lentil breeding program. The breeding program commenced in 1993 with the varieties Cobber, Digger and Matilda released by Agriculture Victoria (Brouwer, 1995a,b; Materne and Brouwer, 1997), and all early introductions such as Eston and Indianhead were given 1993 as their “year of release.” Varieties released after 1993 that can be found in IP Australia’s plant breeder’s rights register^1^ were labelled with the year when the variety was accepted, even if they were officially released to farmers after this time. Lines from the Coordinated Improvement Program for Australian Lentils (CIPAL) were unreleased advanced breeding germplasm possessing key traits in the breeding program; their years of release were estimated based on the number of years they were evaluated under the National Variety Trials (NVT) program as used in previous studies (Lake and Sadras, 2021). Landmark varieties were selected to represent a “historical germplasm set” from 1993 to 2020 (Supplementary Table 1). Four additional lines (Commando, SP1333, ILL2024 and ILL7537) were included due to their parentage relevance in the breeding program. However, these lines were either poorly adapted to Australian conditions or did not have the right trait combinations for variety release.

Two main experiments studying 36 lentil varieties in a randomised block design with three replicates were carried out during 2020 and 2021. In the first field season, twenty traits were investigated that had potential to correlate with the yield increases conferred through breeding. In the second field season the most relevant traits were measured, such as plant height, canopy temperature depression (CTD) and yield components. Trials in both years were sown after a year-long fallow to ensure sufficient soil water for good growth under in season rainfed conditions. Each replicate consisted of two plots side by side with each plot measuring 5 m long × 1.25 m wide, comprised 5 rows on 25 cm spacing. One plot was used for sacrificial biomass harvests throughout the season and the other for grain yield assessments at the end of the season. Trials were sown on 28 th May 2020 and on 4 th June 2021 in the field experimental site from Agriculture Victoria at Horsham, Australia (36°43′40.4′′S 142°05′10.4″E).

### Measured traits

During 2020 and 2021, a range of morphological and physiological traits were measured at vegetative, flowering and podding stages (Table 1). Period of measurement is expressed as days after sowing (DAS) and thermal time is available in Supplementary Table 2.

**Table 1:** Traits measured in 2020 and 2021 (see text for trait definitions).

Traits measured	2020	2021
Yield	✓	✓
Biomass veg, F21, mat	✓	✓
Harvest index (HI)	✓	✓
Canopy temperature depression (CTD) F8, F20, F38, F53	✓	✓
Plant height veg and F21	✓	✓
Pod wall ratio	✓	
Seed size	✓	
Leaf size veg and flw	✓	
Stem dry weight veg and flw	✓	
Leaves dry weight veg and flw	✓	
Plant dry weight veg and flw	✓	
Chlorophyll content veg and flw	✓	
Leaf area per plant veg and flw	✓	
Leaf dry mass per area (LMA) veg and flw	✓	
Number of branches veg and flw	✓	
Plant height veg and flw	✓	
Light interception (*f*APAR*i*) 49,61,89,105,137,180 DAS	✓	
Accumulated photosynthetic active radiation (APAR) flw	✓	
Radiation use efficiency (RUE) veg and flw	✓	
RUE veg to flw	✓	
CO_2_ assimilation rate (*A*) flw and pod	✓	
Stomatal conductance (*g_s_*) flw and pod	✓	
Plant height mat		✓
Canopy height model (CHM) 95, 110, 126, 137, 123, 136, 143, 153, 166 DAS		✓

DAS, days after sowing; veg, vegetative stage; flw, flowering stage; pod, early podding stage; mat, physiological maturity stage; F plus a number indicates the approximate number of days from the first flowering score; F8 (127 DAS in 2020 and 123 DAS in 2021); F21 (138 DAS in 2020 and 137 DAS in 2021); F38 (158 DAS in 2020 and 153 DAS in 2021); F53 (173 DAS in 2020 and 166 DAS in 2021).

### Gas exchange

Assimilation rate (*A*, μmol CO_2_ m^−1^ s^−1^) and stomatal conductance (*g_s_*, mol H_2_O m^−2^ s^−1^) were measured using a LI-COR 6400 infrared gas analyser (LI-COR Inc., Lincoln, NE, USE). The CO_2_ flow rate into the leaf chamber was set at 500 μmol s^−1^, irradiance of 1800 μmol quanta m^−1^ s^−1^, inlet CO_2_ at 400 μmol CO_2_ (mol air)^−1^ and block temperature was 25°C. Three leaflets from the third or fourth expanded leaf from the top of a branch with flowers or pods were fitted into the chamber of the 6 cm^2^ rectangular head. Plants were measured on different days to match similar plant stages. At flowering measurements were done at 130, 131, 137, 138 and 139 DAS. Eston and Indianhead were not measured because they were not flowering at the time of the measurements. At early podding measurements were done at 158, 160, 161, 162, 165, 166, 168 and 169 DAS.

### Canopy temperature depression

In both years, canopy temperature was measured in the field in the non-destructive plots using a hand-held infrared thermometer (Agri-Therm III™ 6210 l; Everest Interscience Inc., United States). Measurements were taken by pointing the thermometer at the plant canopy at about a 45 ° oblique angle, when the sky was clear, medium-low wind speed, and ambient temperature approached the maximum for the day and when it was most constant at four randomly selected positions within each plot during flowering, podding and late podding. CTD was calculated as the difference between air temperature and canopy temperature (Pietragalla, 2012; Mason et al., 2013).

### Plant height, branching, chlorophyll content and leaf area

In 2020, measurements for plant height, total number of branches, chlorophyll content, leaf size and leaf area per plant were collected. Three plants per plot were sampled at vegetative (90 DAS) and flowering (138 DAS) stages and stored at 4°C for up to 5 days before processing. Chlorophyll content was estimated using a SPAD-502PLUS chlorophyll meter (Minolta Camera Co., Ltd., Japan) collected from sampling 10 leaflets per plant and averaged to get a single value for each plant. Leaf size was recorded by passing individual compound leaves from each plant through a leaf area meter (LI-3100C Area Meter) at vegetative stage and 10 leaves per plant at the flowering stage. Leaf area per plant was recorded by passing all leaves from each plant through the leaf area meter. Leaves and stems from each plant were oven dried at 70°C for 72 h separately to obtain their respective dry weight and total plant weight. Leaf dry mass per area (LMA) was calculated from the total leaf area per plant and the total dry weight per plant.

In 2021, plant height was measured manually in the field at maturity and in other plant stages using a canopy height model (CHM) from data collected with an unmanned aerial vehicle (UAV). The aerial images were acquired using a multispectral imager MicaSense RedEdge MX (MicaSense Inc., Seattle, WA, United States) attached to a quadcopter DJI Matrice 210 RTK (DJI Technology Co, Shenzhen, China). All the flight missions were conducted at 30 m above ground level to estimate CHM. The Pix4D mapper (Pix4D, Lausanne, Switzerland) software was used to pre-process the images to obtain a map with a digital surface model (DSM) and a digital terrain model (DTM). The CHM was generated by pixelwise subtraction of DTM from DSM from rectangular polygons with a unique plot number (Gebremedhin et al., 2020).

### Light interception (*f*APAR*i*)

Light interception was assessed during 2020 as the fraction of absorbed photosynthetically active radiation (*f*APAR*i*) measured with a linear ceptometer (AccuPAR LP-80; Decagon Devices, Inc., United States) at vegetative, flowering and podding stages (Table 1). Percentage of *f*APAR*i* was calculated by comparing the incident photosynthetic active radiation (PAR) above canopy (PAR*i*) and below canopy (PAR*g*; Eq. 1).


(1) $$\;f{\mathrm{PAR}}_{i}\;\left(\%\right)=\frac{{\mathrm{PAR}}_{i}-{\mathrm{PAR}}_{g}\;}{{\mathrm{PAR}}_{i}}\times 100$$

### Radiation use efficiency

RUE was calculated using the values of *f*APAR*i*, incident photosynthetic active radiation (PAR) and aboveground biomass. Sigmoidal curves were fitted with GraphPrism Version 9.1.2 (average *R*^2^ = 0.99; RMSE = 4%) to describe the dynamics of *f*APAR*i* from sowing to early podding (153 DAS). Daily APAR was calculated from daily *f*APAR*i* from fitted curves and PAR calculated as half of the total incident solar radiation (Trapani et al., 1992). RUE was calculated as the ratio between biomass and APAR (Verón et al., 2005) for vegetative (RUE veg, 90 DAS) and flowering (RUE flw,138 DAS) stages and for the period between them (RUE veg to flw).

### Yield, biomass and yield components

In both years, the plot layout included two plots located side by side: one for destructive measurements and other for non-destructive measurements. In 2020, samples of aboveground biomass from vegetative (90 DAS), flowering (138 DAS) and physiological maturity stages (197 DAS) were obtained by sampling 0.375 m^2^ from the three middle rows of a destructive plot (0.5 × 0.75 m). Biomass was oven dried at 70°C for 72 h and dry weight was recorded. The yield from the destructive plot was obtained from the biomass cut at maturity, this yield was only used to calculate HI. Fifty random pods were selected to calculate pod wall ratio (Sadras et al., 2013) and 100 seeds were weighted to estimate seed size. In 2021, the biomass was harvested in five rows (0.5 × 1.75 m) at vegetative (95 DAS), flowering (138 DAS) and physiological maturity stages (196 DAS). HI was calculated from the later biomass cut and the yield harvested by machine.

Yield presented in results from both years was sampled from the non-destructive plots and harvested by machine with a Wintersteiger Delta header at physiological maturity, at 202 and 200 DAS in 2020 and 2021, respectively. Area was calculated from the plot length (5 m) multiplied by the distance from plot centre to plot centre (1.75 m) to give a total area per plot of 8.75 m^2^, following the standard measurement in NVT in Australia as it best approximates broad acre yields.

### Statistical analysis

Statistical analyses were undertaken using linear mixed models (LMM) with the software package ASReml-R v4, VSN International in R language (Butler et al., 2017). Each trait from 2020 was analysed individually exploring spatial correlations using R following the steps in the Biometry Training 0.7.1 package (Nielsen et al., 2021) and analysis from the 2021 season was performed using the released version in CRAN ‘Biometryassist 1.0.0’ (Nielsen et al., 2022). The model was fitted using each trait (e.g., plant height) as the response variable, the varieties as the explanatory component, and the experimental unit was plant within plot or plot defined by row and column. DAS was used as random term in the analysis of *A* and *g_s_* to account for the five different days that took to take these measurements. Wald test was used to evaluate significant differences across varieties and years. Prediction of response values were obtained and predicted means were compared between varieties using Tukey’s multiple comparison (*p* ≤ 0.05). Pearson correlation matrices were calculated based on the predicted mean trait values for each variety using the R package ‘agricolae’ (De Mendiburu, 2021) and their detail output are presented in the Supplementary Tables 3–9.

## Results

### Environmental conditions

**Figure 1:**
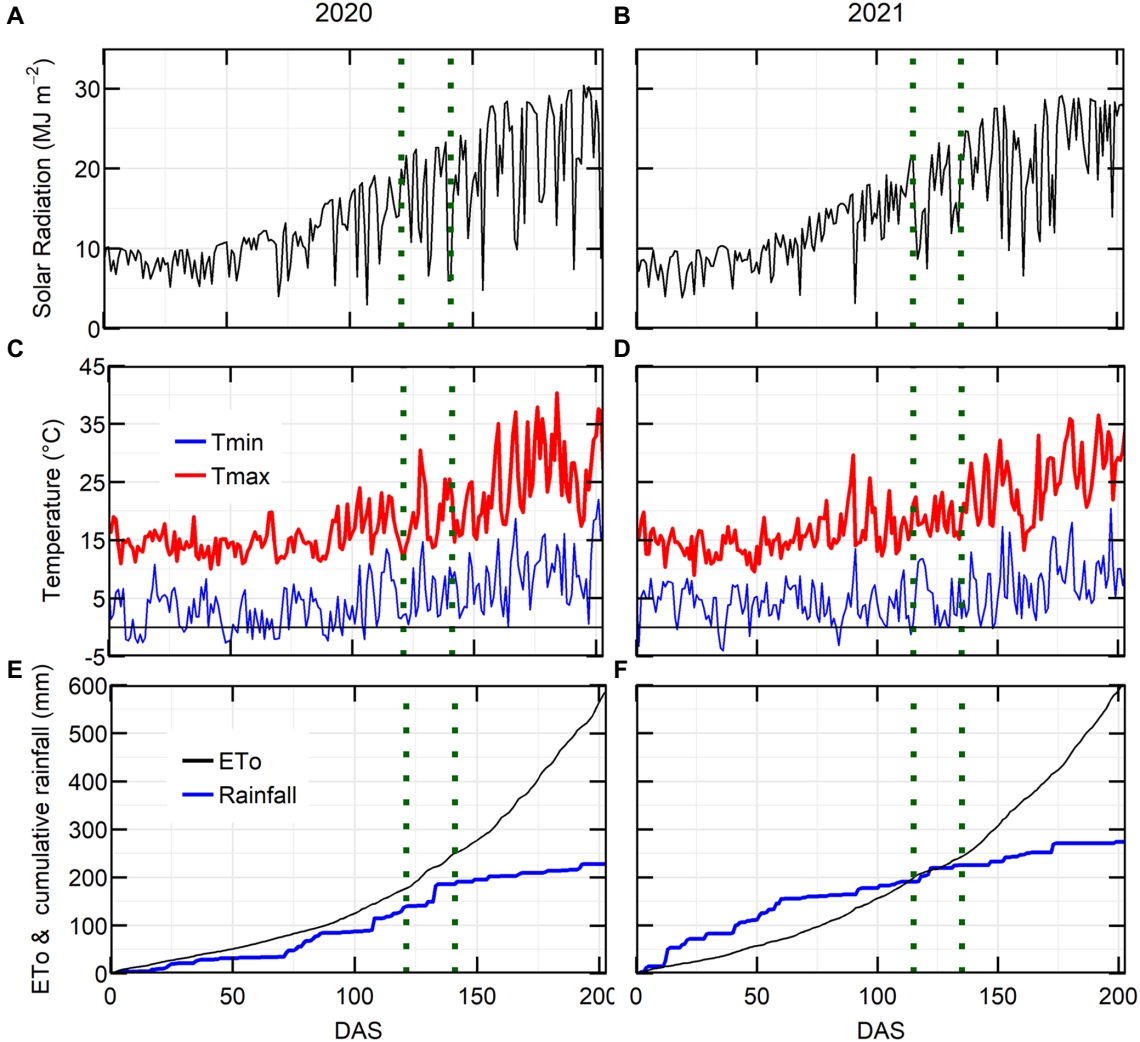
Environmental conditions for lentil crop at Horsham in 2020 and 2021. Solar radiation **(A,B)**, maximum and minimum temperature **(C,D)**, evapotranspiration and cumulative rainfall **(E,F)** in relation to days after sowing (DAS). Flowering window is located in between the dotted green lines.

Growing season conditions were generally favourable in both years, with average grain yield from the latest release variety PBA Kelpie XT ranging from 2.87 (2020) to 2.93 (2021) t ha^−1^. The maximum solar radiation and maximum temperature were higher in 2020 than in 2021 (Figures 1A–D). The 2020 growing season rainfall (214 mm from sowing to November) was drier than 2021 (271 mm of rain). The major rainfall events (20–30 mm) in 2020 occurred during the flowering window, while in 2021 they occurred at crop establishment and late podding (Figures 1E,F).

### Yield components

The statistical analysis showed that variety (genotype, G) was significant for yield, biomass and HI, while year (environment, E) was significant for yield and biomass. There was a significant G × E interaction for yield, biomass F21 (21 days from the first flowering score) and HI (Table 2).

**Table 2:** Value of *p* from yield component traits measured in 2020 and 2021.

Trait	Variety	Year	Variety × Year
Yield	≤0.001^***^	≤0.001^***^	≤0.001^***^
Biomass veg	≤0.001^***^	≤0.001^***^	0.2469^ns^
Biomass F21	≤0.001^***^	≤0.001^***^	≤0.05^*^
Biomass mat	≤0.001^***^	≤0.01^**^	0.4490^ns^
HI	≤0.001^***^	0.0702^ns^	≤0.001^***^

F21, 21 days from the first flowering score (138 DAS in 2020 and 137 DAS in 2021); veg, vegetative stage; mat, Maturity stage. Variety degrees of freedom: 35.

ns *p* > 0.05;

* *p* ≤ 0.05;

** *p* ≤ 0.01;

*** *p* ≤ 0.001.

Average yield in 2021 was 0.68 t ha^−1^ higher than 2020 (Tables 3, 4). In 2020, yield progress increased 0.0328 ± 0.0048 t ha^−1^ year^−1^ or 1.18 ± 0.17% year^−1^ and in 2021 there was a positive increase of 0.0315 ± 0.0045 t ha^−1^ year^−1^ or 1.07 ± 0.16% year^−1^. Average biomass at vegetative, flowering and maturity stages decreased 40, 17 and 15%, respectively, in 2021 in comparison with 2020, and average HI increased 5% in 2021 (Tables 3, 4).

**Table 3:** Average of traits measured in 2020 from the predicted means of linear mixed model and standard error (SE); value of *p* is the genotypic value of *p* from the Wald test.

**Trait**	**Average**	**SE**	**DF**	Value of ***p***	**R YOR**
2020					
Yield (t ha^−1^)	1.91	0.09	35	≤0.001	0.62***
Biomass veg (g m^−2^)	58.03	1.97	35	0.087	0.12^ns^
Biomass flw (g m^−2^)	362.93	7.58	35	≤0.001	0.30^ns^
Biomass mat (g m^−2^)	746.50	17.78	35	0.212	0.38^ns^
HI	0.39	0.01	35	≤0.001	0.49**
Pod wall ratio	6.22	0.14	35	≤0.001	-0.12^ns^
Seed size (g)	3.76	0.16	35	≤0.001	0.15^ns^
Leaf size veg (cm^2^)	1.95	0.07	35	≤0.001	0.35*
Leaf size flw (cm^2^)	6.87	0.18	35	≤0.001	0.28^ns^
Stem dry weight veg (g)	0.08	0.004	35	≤0.010	0.12^ns^
Stem dry weight flw (g)	1.65	0.06	35	0.134	0.38*
Leaves dry weight veg (g)	0.12	0.01	35	≤0.001	0.09^ns^
Leaves dry weight flw (g)	1.23	0.04	35	0.511	0.17^ns^
Plant dry weight veg (g)	0.20	0.01	35	≤0.001	0.11^ns^
Plant dry weight flw (g)	2.88	0.10	35	0.277	0.30^ns^
SPAD veg	39.15	0.48	35	≤0.001	0.23^ns^
SPAD flw	46.48	0.42	35	≤0.001	0.20^ns^
Leaf area per plant veg (cm^2^)	27.66	1.25	35	≤0.010	0.13^ns^
Leaf area per plant flw (cm^2^)	325.56	9.33	35	0.820	0.13^ns^
LMA veg (g m^−2^)	42.69	0.66	35	≤0.050	-0.13^ns^
LMA flw (g m^−2^)	37.94	0.55	35	≤0.001	0.08^ns^
Number of branches veg	2.69	0.12	35	≤0.001	-0.27^ns^
Number of branches flw	8.07	0.32	35	0.107	−0.49**
Plant height veg (cm)	11.18	0.27	35	≤0.001	0.34^ns^
Plant height flw (cm)	38.68	0.84	35	≤0.001	0.73***
*f*APAR*_i_*, 49 DAS (%)	1.31	0.08	35	≤0.001	−0.11^ns^
*f*APAR*_i_*, 61 DAS (%)	2.67	0.21	35	≤0.001	0.11^ns^
*f*APAR*_i_*, 89 DAS (%)	16.74	0.04	35	≤0.001	0.29^ns^
*f*APAR*_i_*, 105 DAS (%)	38.71	0.01	35	≤0.010	0.16^ns^
*f*APAR*_i_*, 137 DAS (%)	93.96	0.10	35	≤0.010	0.45**
*f*APAR*_i_*, 180 DAS (%)	87.34	1.25	35	≤0.050	0.01^ns^
APAR flw (MJ m^−2^)	236.35	5.25	35	≤0.001	0.24^ns^
RUE veg (g MJ^−1^)	0.83	9.33	34	≤0.050	−0.13^ns^
RUE flw (g MJ^−1^)	1.55	0.07	35	≤0.010	0.01^ns^
RUE veg to flw (g MJ^−1^)	1.37	0.18	35	0.223	0.16
*A* flw (μmol CO_2_ m^−2^ s^−1^)	23.29	0.66	34	≤0.050	−0.41*
*A* pod (μmol CO_2_ m^−2^ s^−1^)	12.17	0.55	34	0.626	0.26^ns^
*g_s_* flw (mol H_2_O m^−2^ s^−1^)	0.41	0.48	34	0.129	−0.47**
*g_s_* pod (mol H_2_O m^−2^ s^−1^)	0.09	0.42	34	0.277	0.20^ns^
CTD, 127 DAS (°C)	0.36	0.27	35	≤0.001	−0.47**
CTD, 138 DAS (°C)	−0.22	0.84	35	≤0.001	0.25^ns^
CTD, 158 DAS (°C)	−0.89	0.12	35	≤0.001	−0.33^ns^
CTD, 165 DAS (°C)	−1.86	0.32	35	≤0.010	−0.35^ns^
CTD, 173 DAS (°C)	−4.00	0.08	35	≤0.010	0.01^ns^

R YOR is the Pearson correlation of the trait with year of release (populated from Supplementary Tables 3, 5, 6, 8).

ns *p* > 0.05;

* *p* ≤ 0.05;

** *p* ≤ 0.01;

*** *p* ≤ 0.001.

**Table 4:** Average of traits measured in 2021 from the predicted means of linear mixed model and standard error (SE); value of *p* is the genotypic value of *p* from the Wald test.

Trait	Average	SE	DF	Value of *p*	R YOR
Yield (t ha^−1^)	2.59	0.10	35	≤0.001	0.64***
Biomass veg (g m^−2^)	34.61	1.42	35	≤0.001	0.30^ns^
Biomass flw (g m^−2^)	299.86	10.34	35	≤0.001	0.45**
Biomass mat (g m^−2^)	637.38	18.66	35	≤0.010	0.06^ns^
HI	0.41	0.02	35	≤0.001	0.59***
Plant height mat (cm)	33.15	0.77	35	≤0.001	0.44*
CHM, 95 DAS (cm)	9.06	0.32	35	≤0.001	0.23^ns^
CHM, 110 DAS (cm)	13.25	0.36	35	≤0.001	0.46**
CHM, 126 DAS (cm)	18.57	0.65	35	≤0.001	0.59***
CHM, 137 DAS (cm)	24.08	0.85	35	≤0.001	0.66***
CTD, 123 DAS (°C)	0.68	0.05	35	0.119	−0.09^ns^
CTD, 136 DAS (°C)	−0.34	0.05	35	≤0.050	0.08^ns^
CTD, 143 DAS (°C)	−2.35	0.08	35	≤0.050	0.35*
CTD, 153 DAS (°C)	−1.60	0.06	35	≤0.050	0.31^ns^
CTD, 166 DAS (°C)	−4.49	0.12	35	0.075	−0.10^ns^

R YOR is the Pearson correlation of the trait with year of release (populated from Supplementary Tables 4, 7, 9).^ns^*p* > 0.05; *p ≤ 0.05; **p ≤ 0.01; ***p ≤ 0.001.

**Figure 2:**
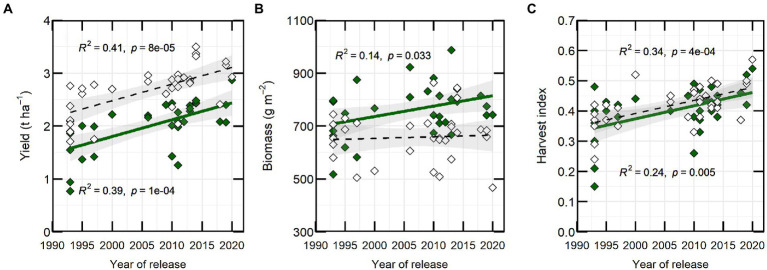
Yield **(A)**, biomass **(B)** and harvest index **(C)** versus year of release from 32 varieties of lentils from 2020 (closed-green symbol) and 2021 (open symbol). Each symbol is the predicted mean of three repetitions. Line fits and coefficients of determination (*R^2^*) are shown for significant relationships.

Year of variety release correlated positively with yield in both years, with a coefficient of determination of approximately 0.4 (Figure 2A). Varieties released from 2018 to 2020 (PBA Hallmark XT, PBA Highland XT and PBA Kelpie XT) yielded from 0.63 (in 2021) to 0.83 t ha^−1^ (in 2020) more than initial varieties from 1993 (Eston, Indianhead, Cobber, Digger and Matilda). Aboveground biomass at maturity was weakly correlated with year of release (YOR) in 2020 (*R^2^* = 0.14; *p* ≤ 0.05) but not in 2021 (Figure 2B). HI increased 0.43% with YOR and correlated positively with YOR (*R^2^* ~ 0.3; *p* ≤ 0.05; Figure 2C).

### Plant architecture and partitioning changed as consequence of breeding

In 2020, physiological and morphological traits were assessed at different growth stages to identify traits that changed with the year of variety release. At vegetative stage: leaf size; and at flowering stage: stem dry weight, plant height, number of branches and *f*APAR*i* (137 DAS) had a significant correlation with YOR (Table 3).

**Figure 3:**
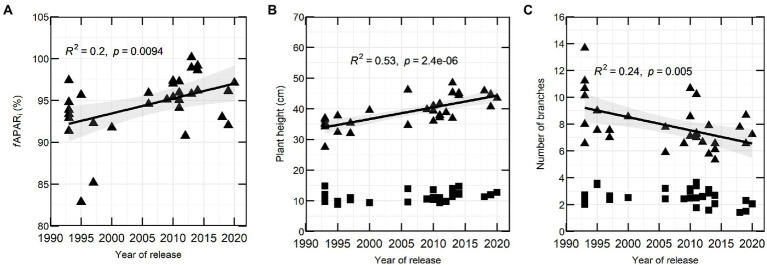
Percentage of *f*APAR*i* **(A)**, plant height **(B)** and number of branches **(C)** in 32 lentil varieties in relation to YOR at vegetative stage (squares) and flowering stage (triangles) measured during 2020 field season.

In 2020, *f*APAR*i* correlated significantly with YOR at flowering (137 DAS; Figure 3A). There were also marked differences in canopy architecture and YOR, with recently released varieties having increased height and reduced total number of branches at flowering (Figures 3B,C).

Because of significant positive correlation of *f*APAR*i* at 61, 89, 105 and 137 DAS with plant height (Supplementary Table 6) and the ability to estimate plant height (canopy height model, CHM) from an UAV, it was decided to measure plant height in the second year. In 2021, CHM measured between 1 week before flowering and late flowering (110–137 DAS) was positively correlated with YOR (Table 4).

Analysing the 2 years of measurement, plant height was significant different across years and across genotypes (Table 5). Despite G × E effects observed at flowering, it is possible to observe that there is generic variation across genotypes when they are analysed by year (Tables 3, 4), which support the CHM measurements in the breeding program.

**Table 5:** Wald test for plant height and canopy temperature depression (CTD) measured in 2020 and 2021.

Trait	Variety	Year	Variety × Year
Plant height veg	≤0.001^***^	≤0.001^***^	0.0814^ns^
Plant height F21	≤0.001^***^	≤0.001^***^	≤0.001^***^
CTD F8	≤0.001^***^	0.4803^ns^	≤0.01**
CTD F20	≤0.001^***^	0.7482^ns^	≤0.05*
CTD F38	≤0.001^***^	0.4796^ns^	≤0.001^***^
CTD F53	≤0.001^***^	0.6110^ns^	0.0629^ns^
CTD Avg	≤0.001^***^	0.4753^ns^	≤0.001^***^

F and a number indicates the approximate number of days from the first flowering score; F8 (127 DAS in 2020 and 123 DAS in 2021); F21 (138 DAS in 2020 and 137 DAS in 2021); F38 (158 DAS in 2020 and 153 DAS in 2021); F53 (173 DAS in 2020 and 166 DAS in 2021).

ns *p* > 0.05;

* *p* ≤ 0.05;

** *p* ≤ 0.01;

*** *p* ≤ 0.001.

### Historical and genetic variation in CO_2_ assimilation, stomatal conductance and canopy temperature depression

**Figure 4:**
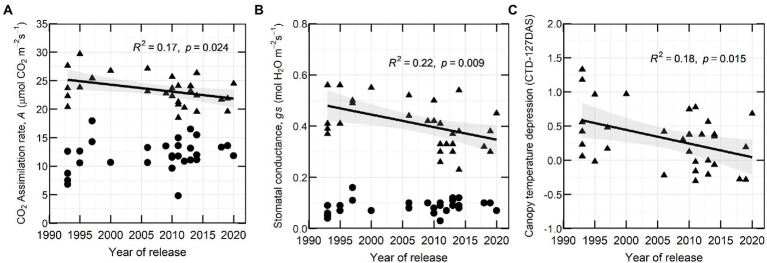
**(A)** CO_2_ assimilation rate (*A*), **(B)** stomatal conductance (*g_s_*) and **(C)** canopy temperature depression at 127 DAS in relation with year of release (YOR), at flowering (triangles) and early podding stages (circles) from 30 historical lentil varieties in 2020.

In 2020, CO_2_ assimilation rate (*A*) and stomatal conductance (*g_s_*) at flowering decreased with YOR (*R*^2^ of 0.17 and 0.22 respectively) and there was no clear association between these traits and YOR of the varieties at early podding (Figures 4A,B). Canopy temperature depression (CTD) at early flowering (127 DAS) showed a similar trend with a negative correlation to YOR (Figure 4C).

CTD variation across the 2 years was mostly attributed to the varieties. There was a G × E interaction for most of the point measurements, but there were no significant differences across years (Table 5).

**Figure 5:**
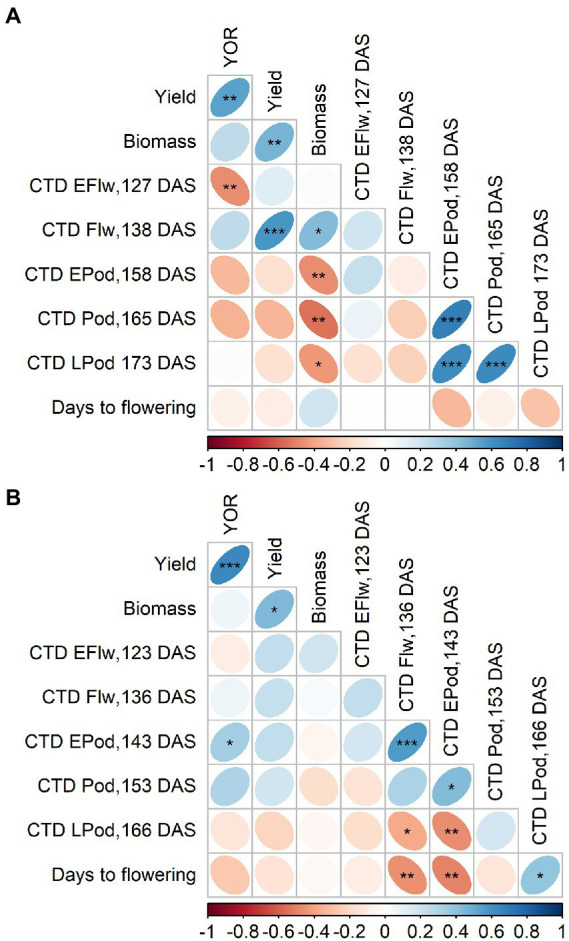
Pearson correlation from 2020 **(A)** and 2021 **(B)** of year of release (YOR), yield, biomass and canopy temperature depression (CTD) at early flowering (EFlw), flowering (Flw), early podding (EPod), podding (Pod) and late podding (LPod). Plotted from Supplementary Tables 8, 9.

There were changes in polarity of the correlation between CTD and YOR. In 2020, this relationship was positive at 138 DAS, and returned to negative at 127, 158 and 165 DAS. At 138 DAS, CTD also positively correlated with yield and with biomass while negative correlations were observed at 158, 165 and 173 DAS (Figure 5A).

Although most of the correlations in 2021 were not significant, CTD and YOR relationship was positive at 143 and 153 DAS (Figure 5B). At 123, 136, 143 and 153 DAS, CTD positively correlated with yield (Figure 5B).

## Discussion

Our study from 2 years data indicated a lentil yield increase of 1.1% *per annum*. This is in agreement with previously reported by Sadras et al. (2021) at Roseworthy, South Australia and by Bogale et al. (2017), in Ethiopia. Our data support the previous studies, but also indicates some changes in morpho-physiological traits related to radiation capture and photosynthesis. Most of these traits were assessed in 2020 and few of them were selected to be measured again in 2021 (Table 1); from these initial results, we observed that canopy architecture (height, branch number, leaf size and intercepted radiation) were associated with the year of release. Breeders currently assess plant architecture *via* measuring plant height and growth habit (erectness or sprawling), which in turn has resulted in selection for branch number and light interception. Our preliminary studies suggest an indirect selection for lower CO_2_ assimilation rate and stomatal conductance, which were lower in recently released varieties in comparison with early released varieties. However, further studies in multiple environments need to be done to confirm these observations.

Genetic yield increases have also been related to phenology, particularly in environments experiencing severe water stress with yield ≤560 kg ha^−1^ as indicate by Lake and Sadras (2021) and reinforces our study. New lentil cultivars adapted for Australia have earlier flowering and longer grain filling periods than older cultivars (Lake and Sadras, 2021; Sadras et al., 2021). Our study also supports the increase in yield gains. All this advances in grain yield numerical components and biomass, were also reflected in HI as previously reported (Lake and Sadras, 2021).

### Crop architecture

Plant height and branch number are the traits that have undergone the largest change over this period shown by a strong RYOR of 0.73 and −0.49, respectively, (Table 3). A long-term goal in lentil breeding has been to include plant height and erectness in selection programs so as to enable the use of machinery for harvest (Erskine et al., 1988). The importance of plant height has also been observed in 287 diverse lentil accessions where plant height correlated with biomass and yield (Tullu et al., 2001). Similarly, in the Australian lentil breeding program, preliminary results from this work showed that higher yielding varieties were taller with fewer branches than the low yielding varieties. This could indicate that instead of producing more branches, recently released varieties allocate more carbon resources to their grains. Although we did not measure carbohydrates in this study, evidence in soybean indicates that changes in carbon economy (storage and partitioning) could support the higher yields of new cultivars (Morrison et al., 1999).

Plant height at flowering in 2020 correlated positively with light interception and HI (Supplementary Table 6). A remotely sensed measurement is faster and more convenient than manually screen many lines for this trait. In 2021, plant height was assessed as CHM, which is an estimation of crop height using UAV imagery (Banerjee et al., 2020). CHM correlated positively with YOR and yield mostly at flowering, suggesting that this trait can be assessed using field-based phenotyping remote sensing as has been also done recently in field peas (Tefera et al., 2022). Lentil plant height is even more important under stressful conditions, as it tends to be reduced in such environments (Lake and Sadras, 2021) and assessing plant height with remote sensing can be an efficient alternative for a breeding program.

### Genotypic diversity in light interception

Our work indicated genotypic diversity for the proportion of light intercepted at flowering. Interestingly, there was a wide genotypic diversity in *f*APAR*_i_* in all points measured covering from vegetative to flowering stages, and at flowering, it varied up to 25% across the genotypes assessed (Table 3). These results might differ with environment since we only measured 1 year, but similar genotypic variation also has been observed for light interception in lentils and chickpeas (Hanlan et al., 2006; Li et al., 2008). There were also differences across pulses, in a study comparing chickpea, lentil, narrow-leafed lupin and field pea found that narrow-leafed lupin intercepted more radiation than the other pulse species (Ayaz et al., 2004). This shows that across pulses and within lentils, there is genotypic variation for light interception that can be exploited.

In our study, both *f*APAR*i* and RUE from vegetative to flowering (RUE veg to flw) were positively correlated with yield (Supplementary Table 6). From the equation Yield = LI*RUE*HI (Reynolds et al., 2009), LI and RUE both contribute to yield and there appears to be an opportunity in lentils for improvement in both factors. Light conversion efficiency represented by RUE has been proposed to be a major trait for genetic improvement since most crops use only 12% of the total solar radiation (Reynolds et al., 2000b). For chickpeas, greater conversion of sunlight into biomass (higher RUE) has been observed at high plant population densities (400 plants m^−2^; Ayaz et al., 2004). In lentils it has been suggested that canopy closure at an early stage increases the time available to produce greater biomass (McKenzie and Hill, 1991). Taller genotypes have shown the highest percentage of *f*APAR*i* in lentils (based on the significative correlation between plant height and *f*APAR*i*, Supplementary Table 6). However, a lentil genotype with slightly less biomass, plant height and branch number had the highest HI and yield (Hanlan et al., 2006).

The future Australian lentil breeding program focus might be towards continue developing varieties with moderate plant height and number of branches with relatively higher light interception and RUE without any or minimal penalty in HI focusing on reproductive nodes that increase sink strength (Whitehead et al., 2000; Bourgault et al., 2017).

### Assessing CO_2_ assimilation rate, stomatal conductance and canopy temperature

Gas exchange traits were only measured 1 year, which can be subject to G × E interaction. Our preliminary results indicate that *A* and *g_s_* measured at flowering tended to be lower in recently released varieties compared to those released at the commencement of the Australian breeding program, particularly some of the varieties released since 2011. These results differ from observations in wheat, soybean and dry beans, where recently released cultivars or high yielding cultivars had the highest photosynthetic rate, daily carbon uptake or stomatal conductance (Peet et al., 1977; Fischer et al., 1998; Morrison et al., 2000; Jin et al., 2010; Liu et al., 2012; Koester et al., 2016) and findings in wheat where CO_2_ assimilation rate did not change over five decades of breeding in Australia (Sadras et al., 2012). Reduced stomatal conductance in legumes has been related to an increase of water use efficiency (WUE; Adams et al., 2018). The reduced *A* and *g_s_* observed in recently released varieties from 1 year experiment presumably is related to WUE. Although, we did not study WUE, there is a possibility of an indirect selection of new varieties with greater WUE because of the target of the breeding program to expand lentil production in dry areas (Siddique et al., 2013).

CTD was measured 2 years, in 2020 at 127 DAS had a positive correlation with *g_s_* (Supplementary Table 8) which indicates that could be used as surrogate for *g_s_* as it has been used in different crops (Amani et al., 1996; Deery and Jones, 2021). In 2020 we observed negative correlations between CTD and YOR (Figure 5). However, we did not observe the same pattern in one measurement in 2020 and in the measurements from 2021. After looking at possible environmental factors (Figure 1) that could influence CTD. We arrive to the conclusion that the positive but overall poor correlations between CTD and YOR might be attributed to ambient temperature. Maximum daily temperature on the day of the measurements when we observed positive correlations ranged between 18 and 26°C. While negative correlations between CTD and YOR were detected when ambient temperatures were between 30 and 32°C. This indicates that recently released varieties exhibit cooler canopies when ambient temperature is cooler than 26°C, but at higher temperatures (~30°C), they show a warmer canopy in comparison with early released varieties. This mechanism also points to increased WUE in recently released varieties when are exposed to heat waves of ~30°C. The significant genotypic variation of CTD (Tables 3, 4) aligns with other studies in lentils (Biju et al., 2018) and it is encouraging as there is valuable material to exploit in a breeding program for different environments. Breeding for crop resilience, particularly in a warming world requires to exploit wisely existing or new genetic resources to improve food security (Sharwood et al., 2022).

## Conclusion

We identified that crop architecture has played a key role in yield gains in the Australian National lentil breeding program over the last 27 years. Recently released varieties tend to have a more upright architecture than early released varieties. This has resulted in changes to several measurable traits including plant height, branch number, leaf size and light interception. In addition, photosynthetic traits and canopy temperature depression were measured indicating that recently released varieties are more responsive with stomatal closure at high temperatures. New knowledge of these selected morphological and physiological traits provides an opportunity to assist breeding programs in developing varieties with increased yield and adaptation to Australian cropping systems.

## Data availability statement

The original contributions presented in the study are included in the article/Supplementary material, further inquiries can be directed to the corresponding authors.

## Author contributions

VS-P, ASKS, and SR contributed to the planning and performing of the research. CMC calculated RUE and APAR. GR secured the funding and contributed to the planning. VS-P analysed and wrote the manuscript with contribution from all authors. ATT, GJF, RA, and GMR contributed to manuscript editing. All authors contributed to the article and approved the submitted version.

## Funding

This research was funded by the Victorian Grains Innovation Partnership in a collaboration between Agriculture Victoria and the Grains Research Development Corporation grant ID GRDC 8049295 (VGIP1B).

## Conflict of interest

VS-P is employed by Agriculture Victoria Research in a partnership with the Grains Research Development Corporation. The remaining authors declare that the research was conducted in the absence of any commercial or financial relationships that could be construed as a potential conflict of interest.

## Publisher’s note

All claims expressed in this article are solely those of the authors and do not necessarily represent those of their affiliated organizations, or those of the publisher, the editors and the reviewers. Any product that may be evaluated in this article, or claim that may be made by its manufacturer, is not guaranteed or endorsed by the publisher.

## Footnotes

^1^ https://www.ipaustralia.gov.au/plant-breeders-rights
