# Supplementary material for: Breeding has selected for architectural and photosynthetic traits in lentils
Source: Front Plant Sci. 2022 Aug 25;13:925987. doi: 10.3389/fpls.2022.925987 (PMC9453451; doi:10.3389/fpls.2022.925987)
Supplement: Supplementary file 1 [file Data_Sheet_1.docx]

## **Supplementary tables**

# **Breeding has indirectly selected for architectural and photosynthetic traits in lentils**

Viridiana Silva-Perez, Arun S.K. Shunmugam, Shiwangni Rao, C. Mariano Cossani, Abeya T. Tefera, Glenn J. Fitzgerald, Roger Armstrong and Garry M. Rosewarne

**Supplementary Table 1.** Lentil genotypes (*Lens culinaris*) used in the experiments. The first 32 varieties are “the historical germplasm set”, released between 1993 and 2020 and four are complementary lines.

| No. | Variety | Year/Description | No. | Variety | Year/Description |
| --- | --- | --- | --- | --- | --- |
| 1 | ESTON | 1993 | 19 | MATERNO | 2011 |
| 2 | INDIANHEAD | 1993 | 20 | MT BYRON | 2011 |
| 3 | COBBER | 1993 | 21 | PBA HERALD XT | 2011 |
| 4 | DIGGER | 1993 | 22 | PBA HURRICANE XT | 2012 |
| 5 | MATILDA | 1993 | 23 | PBA ACE | 2013 |
| 6 | PRECOZ | 1993 | 24 | PBA BOLT | 2013 |
| 7 | ALDINGA | 1995 | 25 | CIPAL0901 | 2013 |
| 8 | NORTHFIELD | 1995 | 26 | PBA GIANT | 2014 |
| 9 | CASSAB | 1997 | 27 | PBA GREENFIELD | 2014 |
| 10 | CUMRA | 1997 | 28 | PBA JUMBO2 | 2014 |
| 11 | NUGGET | 2000 | 29 | PBA HALLMARK XT | 2018 |
| 12 | BOOMER | 2006 | 30 | PBA HIGHLAND XT | 2019 |
| 13 | NIPPER | 2006 | 31 | CIPAL1522 | 2019 |
| 14 | PBA FLASH | 2009 | 32 | PBA KELPIE XT | 2020 |
| 15 | CDC RUBY | 2010 | 33 | ILL2024 | International germplasm |
| 16 | PBA BLITZ | 2010 | 34 | ILL7537 | International germplasm |
| 17 | PBA JUMBO | 2010 | 35 | SP1333 | International germplasm |
| 18 | GRAMPIANS | 2011 | 36 | COMMANDO | International germplasm |

**Supplementary Table 2.** Phenological stages and thermal time during the measurements in 2020 and 2021. Day of flowering is the day from when the first genotypes started to flower. Thermal time was calculated by: (T_max_ + T_min_)/2, where T = Daily temperature (°C), using a base temperature of zero. Daily thermal time was summed to derive the accumulated temperature degrees.

| Sowing date |  | 28^th^, May 2020 |  |  |  | 4^th^, June 2021 |  |
| --- | --- | --- | --- | --- | --- | --- | --- |
| Date of the first flowering score |  | 26^th^, Sep 2020 |  |  |  | 27^th^, Sep 2020 |  |
| Stage | **DAS** | **Day of flowering** | **°Cd** |  | **DAS** | **Day of flowering** | **°Cd** |
| Vegetative | 49  61  89  90  105 |  | 420  510  729  736  890 |  | 95  110 |  | 920  1077 |
| Flowering | 127  130  131  137  138  139 | 7  10  11  17  18  19 | 1134  1184  1193  1265  1280  1295 |  | 123  126  136  137  138 | 9  12  22  23  24 | 1236  1268  1377  1388  1402 |
| Early to mid-podding | 153  158  160  161  162  165  166  168  169  173  180 | 33  38  40  41  42  45  46  48  49  53  60 | 1485  1562  1603  1614  1625  1679  1703  1741  1756  1828  1976 |  | 143  153  166 | 29  39  52 | 1473  1628  1800 |
| Final maturity | 197  202 | 77  82 | 2282  2408 |  | 196  200 | 82  86 | 2356  2435 |

DAS: Days after sowing

**Supplementary Table 3.** Pearson correlation of year of release (YOR), yield, biomass cut at vegetative, flowering and maturity stages, harvest index (HI), pod wall ratio and 100 seed weight from 2020, df=30.

|  |  |  | **2020** |  |  |  |  |
| --- | --- | --- | --- | --- | --- | --- | --- |
|  | **YOR** | **Yield** | **Biomass veg** | **Biomass flw** | **Biomass** | **HI** | **Pod wall ratio** |
| **Yield** | **0.62**  *** |  |  |  |  |  |  |
| **Biomass veg** | **0.12**  0.5108 | **0.14**  0.4500 |  |  |  |  |  |
| **Biomass flw** | **0.3**  0.0915 | **0.57**  *** | **0.55**  ** |  |  |  |  |
| **Biomass maturity** | **0.38**  * | **0.61**  *** | **0.32**  0.0756 | **0.46**  ** |  |  |  |
| **Harvest index** | **0.49**  ** | **0.66**  *** | **0.06**  0.76446 | **0.4**  * | **0.35**  0.0516 |  |  |
| **Pod wall ratio** | **-0.12**  0.5152 | **0.37**  0.0364 | **0.13**  0.4709 | **0.1**  0.5998 | **0.18**  0.3147 | **0.38**  * |  |
| **Seed size** | **0.15**  0.4139 | **0.58**  *** | **0.31**  0.0895 | **0.6**  *** | **0.39**  * | **0.43**  * | **0.15**  0.3971 |

**Supplementary Table 4.** Pearson correlation of year of release (YOR), yield, biomass cut at vegetative, flowering and maturity stages and harvest index (HI) from 2021, df=30.

|  |  | **2021** |  |  |  |
| --- | --- | --- | --- | --- | --- |
| Trait | **YOR** | **Yield** | **Biomass veg** | **Biomass flw** | **Biomass** |
| **Yield** | **0.64**  *** |  |  |  |  |
| **Biomass veg** | **0.3**  0.0953 | **0.53**  ** |  |  |  |
| **Biomass flw** | **0.45**  ** | **0.65**  *** | **0.49**  ** |  |  |
| **Biomass maturity** | **0.06**  0.7423 | **0.45**  * | **0.41**  * | **0.25**  0.1631 |  |
| **Harvest index** | **0.59**  *** | **0.67**  *** | **0.14**  0.4303 | **0.4**  * | **-0.31**  0.817 |

**Supplementary Table 5**. Pearson correlation of YOR, Yield, Harvest (HI), stems, leaves and plant dry weight (DW), SPAD, leaf area (LA) per plant, leaf dry mass per area (LMA), leaf size and number of branches measured at vegetative (veg), flowering (flw) stages in the 2020 field season, df=32.

|  |  |  |  |  |  |  |  | **2020** |  |  |  |  |  |  |  |  |  |  |
| --- | --- | --- | --- | --- | --- | --- | --- | --- | --- | --- | --- | --- | --- | --- | --- | --- | --- | --- |
| **Trait** | **YOR** | **Yield** | **HI** | **DW stems veg** | **DW stems flw** | **DW leaves veg** | **DW leaves flw** | **DW plant veg** | **DW plant flw** | **SPAD veg** | **SPAD flw** | **LA per plant veg** | **LA per plant flw** | **LMA veg** | **LMA flw** | **Leaf size veg** | **Leaf size flw** | **Branch veg** |
| **Yield** | **0.62**  *** |  |  |  |  |  |  |  |  |  |  |  |  |  |  |  |  |  |
| **HI** | **0.49**  ** | **0.66**  *** |  |  |  |  |  |  |  |  |  |  |  |  |  |  |  |  |
| **DW stems veg** | **0.12**  0.5101 | **0.19**  0.2923 | **-0.05**  0.7652 |  |  |  |  |  |  |  |  |  |  |  |  |  |  |  |
| **DW stems flw** | **0.38**  * | **0.31**  0.0837 | **0.25**  0.1686 | **0.48**  ** |  |  |  |  |  |  |  |  |  |  |  |  |  |  |
| **DW leaves veg** | **0.09**  0.6248 | **0.09**  0.6111 | **-0.04**  0.8106 | **0.85**  *** | **0.3**  0.0973 |  |  |  |  |  |  |  |  |  |  |  |  |  |
| **DW leaves flw** | **0.17**  0.3567 | **0.17**  0.3542 | **0.11**  0.5572 | **0.53**  ** | **0.94**  *** | **0.36**  * |  |  |  |  |  |  |  |  |  |  |  |  |
| **DW plant veg** | **0.11**  0.5557 | **0.14**  0.4291 | **-0.05**  0.7989 | **0.95**  *** | **0.4**  * | **0.97**  *** | **0.45**  *** |  |  |  |  |  |  |  |  |  |  |  |
| **DW plant flw** | **0.3**  0.0899 | **0.26**  0.1500 | **0.2**  0.2804 | **0.51**  ** | **0.99**  *** | **0.32**  0.0726 | **0.97**  *** | **0.42**  * |  |  |  |  |  |  |  |  |  |  |
| **SPAD veg** | **0.23**  0.2079 | **0.65**  *** | **0.33**  0.0617 | **0.46**  ** | **0.37**  * | **0.37**  * | **0.34**  0.0593 | **0.43**  * | **0.36**  * |  |  |  |  |  |  |  |  |  |
| **SPAD flw** | **0.2**  0.2826 | **0.62**  *** | **0.52**  ** | **0.31**  0.0825 | **0.46**  ** | **0.23**  0.2151 | **0.46**  ** | **0.28**  0.1189 | **0.46**  ** | **0.68**  *** |  |  |  |  |  |  |  |  |
| **LA per plant veg** | **0.13**  0.4666 | **0.15**  0.4062 | **-0.07**  0.7217 | 0.9  *** | **0.33**  0618 | **0.91**  *** | **0.38**  * | **0.94**  *** | **0.36**  * | **0.42**  * | **0.2**  0.2674 |  |  |  |  |  |  |  |
| **LA per plant flw** | **0.13**  0.4841 | **0.09**  0.6121 | **0**  0.9912 | **0.38**  * | **0.86**  *** | **0.27**  0.1397 | **0.89**  *** | **0.33**  0.0676 | **0.88**  *** | **0.26**  0.1521 | **0.34**  0.0540 | **0.29**  0.1090 |  |  |  |  |  |  |
| **LMA veg** | **-0.13**  0.4808 | **-0.04**  0.8222 | **-0.06**  0.7641 | **0.41**  * | **0.15**  0.4154 | **0.62**  *** | **0.21**  0.2461 | **0.55**  ** | **0.17**  0.3655 | **0.2**  0.2752 | **0.2**  0.2722 | **0.35**  0.0527 | **0.17**  0.3529 |  |  |  |  |  |
| **LMA flw** | **0.08**  0.6598 | **0.21**  0.2580 | **0.33**  0.0637 | **0.31**  0.0802 | **0.25**  0.1709 | **0.19**  0.2914 | **0.31**  0.0869 | **0.26**  0.1517 | **0.27**  0.1327 | **0.19**  0.2959 | **0.33**  0.0685 | **0.17**  0.3534 | **-0.14**  0.4487 | **0.13**  0.4654 |  |  |  |  |
| **Leaf size veg** | **0.35**  * | **0.35**  * | **0**  0.9863 | **0.81**  *** | **0.39**  * | **0.78**  *** | **0.4**  * | **0.82**  *** | **0.4**  * | **0.54**  ** | **0.19**  0.2878 | **0.85**  *** | **0.28**  0.1212 | **0.26**  0.1498 | **0.25**  0.1733 |  |  |  |
| **Leaf size flw** | **0.28**  0.1175 | **0.38**  * | **0.1**  0.5815 | **0.23**  0.2143 | **0.37**  * | **0.34**  0.0560 | **0.38**  * | **0.3**  0.0953 | **0.38**  * | **0.45**  * | **0.34**  0.0605 | **0.31**  0.0847 | **0.42**  * | **0.12**  0.5057 | **-0.02**  0.9350 | **0.51**  ** |  |  |
| **Branches veg** | **-0.27**  0.1293 | **-0.3**  0.0971 | **-0.19**  0.2997 | **0.38**  * | **-0.09**  0.6201 | **0.45**  ** | **-0.02**  0.8941 | **0.44**  * | **-0.07**  0.7175 | **-0.04**  0.8456 | **-0.01**  0.9755 | **0.46**  ** | **0.04**  0.8466 | **0.3**  0.0991 | **-0.23**  0.2027 | **0.01**  0.9422 | **-0.24**  0.1821 |  |
| **Branches flw** | **-0.49**  ** | **-0.68**  *** | **-0.45**  * | **-0.02**  0.9234 | **0.2**  0.2738 | **-0.08**  0.6704 | **0.32**  0.0754 | **-0.06**  0.7594 | **0.25**  0.1721 | **-0.29**  0.1030 | **-0.28**  0.1214 | **-0.09**  0.6294 | **0.43**  * | **0.09**  0.6370 | **-0.17**  0.3393 | **-0.22**  0.2169 | **-0.33**  0.643 | **0.21**  0.2449 |

**Supplementary Table 6**. Pearson correlation of YOR, Yield, Biomass, Harvest Index (HI), plant height, light interception (*f*PAR_i_) and Radiation Use Efficiency (RUE) at vegetative (veg) and flowering stages, in between vegetative and flowering stages (RUE veg to flw) and days to flowering in 2020 field season, df=32.

|  |  |  |  |  |  |  | **2020** |  |  |  |  |  |  |  |  |  |
| --- | --- | --- | --- | --- | --- | --- | --- | --- | --- | --- | --- | --- | --- | --- | --- | --- |
| **Trait, DAS** | **YOR** | **Yield** | **Biomass** | **HI** | **Plant height veg** | **Plant height flw** | ***f*PAR_i_ 49** | ***f*PAR_i_ 61** | ***f*PAR_i_ 89** | ***f*PAR_i_ 105** | ***f*PAR_i_ 137** | ***f*PAR_i_ 180** | **APAR Flw** | **RUE veg** | **RUE flw** | **RUE veg to flw** |
| **Yield** | **0.62**  *** |  |  |  |  |  |  |  |  |  |  |  |  |  |  |  |
| **Biomass** | **0.38**  * | **0.61**  *** |  |  |  |  |  |  |  |  |  |  |  |  |  |  |
| **HI** | **0.49**  ** | **0.66**  *** | **0.35**  0.0516 |  |  |  |  |  |  |  |  |  |  |  |  |  |
| **Plant height veg** | **0.34**  0.0558 | **0.46**  ** | **0.34**  0.0603 | **0.05**  0.7685 |  |  |  |  |  |  |  |  |  |  |  |  |
| **Plant height flw** | **0.73**  *** | **0.7**  *** | **0.74**  *** | **0.41**  * | **0.55**  ** |  |  |  |  |  |  |  |  |  |  |  |
| ***f*PAR_i_, 49** | **-0.11**  0.5562 | **-0.14**  0.4519 | **0.02**  0.9031 | **-0.17**  0.3584 | **0.21**  0.2389 | **-0.01**  0.9737 |  |  |  |  |  |  |  |  |  |  |
| ***f*PAR_i_, 61** | **0.11**  0.5487 | **0.18**  0.3216 | **0.12**  0.5211 | **-0.02**  0.9100 | **0.48**  ** | **0.2**  0.2807 | **0.48**  ** |  |  |  |  |  |  |  |  |  |
| ***f*PAR_i_, 89** | **0.29**  0.1129 | **0.36**  * | **0.4**  * | **0.13**  0.4907 | **0.48**  ** | **0.48**  ** | **0.15**  0.4252 | **0.54**  ** |  |  |  |  |  |  |  |  |
| ***f*PAR_i_, 105** | **0.16**  0.3705 | **0.17**  0.3663 | **0.27**  0.1397 | **0.12**  0.5229 | **0.38**  * | **0.32**  0.0702 | **0.05**  0.7709 | **0.39**  * | **0.57**  *** |  |  |  |  |  |  |  |
| ***f*PAR_i_, 137** | **0.45**  ** | **0.5**  ** | **0.5**  ** | **0.05**  0.7975 | **0.53**  ** | **0.54**  ** | **-0.12**  0.5148 | **0.26**  0.1544 | **0.47**  ** | **0.33**  0.0626 |  |  |  |  |  |  |
| ***f*PAR_i_, 180** | **0.01**  0.9648 | **0.13**  0.4649 | **0.2**  0.2833 | **0.01**  0.9537 | **0.24**  0.1919 | **0.27**  0.1355 | **0.21**  0.2391 | **0.12**  0.5036 | **0.19**  0.3049 | **0.05**  0.7682 | **0.21**  0.2539 |  |  |  |  |  |
| **APAR Flw** | **0.24**  0.1853 | **0.35**  * | **0.43**  * | **0.1**  0.5935 | **0.48**  ** | **0.44**  * | **0.15**  0.4051 | **0.53**  ** | **0.74**  *** | **0.83**  *** | **0.63**  *** | **0.25**  0.1653 |  |  |  |  |
| **RUE veg** | **-0.13**  0.4815 | **-0.33**  0.0671 | **-0.16**  0.3854 | **-0.13**  0.4924 | **-0.5**  ** | **-0.22**  0.2227 | **-0.05**  0.8065 | **-0.48**  ** | **-0.55**  ** | **-0.34**  0.0565 | **-0.25**  0.1735 | **-0.28**  0.1212 | -0.53 |  |  |  |
| **RUE flw** | **0.01**  0.9354 | **0.14**  0.4298 | **-0.07**  0.7222 | **0.32**  0.0704 | **-0.14**  0.4403 | **0**  0.9915 | **0.03**  0.8687 | **-0.13**  0.4886 | **-0.09**  0.6375 | **-0.59**  *** | **-0.07**  0.6836 | **0.17**  0.3577 | -0.43 | **0.16**  0.3853 |  |  |
| **RUE**  **veg to flw** | **0.16**  0.3800 | **0.37**  * | **0.12**  0.5012 | **0.42**  * | 0.04  0.8218 | **0.24**  0.1893 | **0.07**  0.6968 | **0.05**  0.7972 | **0.25**  0.1594 | **-0.31**  0.0860 | **0**  0.9917 | **0.12**  0.4966 | -0.11 | **-0.05**  0.7877 | **0.77**  *** |  |
| **Days to flw** | **-0.31**  0.0877 | **-0.48**  ** | **-0.18**  0.3286 | **-0.75**  *** | -0.26  0.1521 | **-0.29**  0.1047 | **0.21**  0.2577 | **0.03**  0.8532 | **-0.17**  0.3391 | **-0.25**  0.1656 | **0.02**  0.9280 | **-0.03**  0.8742 | -0.21 | **0.37**  * | **-0.12**  0.4977 | **-0.3**  0.0957 |

**Supplementary Table 7**. Pearson correlation of YOR, Yield, Biomass, Harvest Index (HI), canopy height model (CHM) at 95, 110, 126 and 137 days after sowing (DAS) and plant height at maturity from 2021 field season, df=32.

|  |  |  | **2021** |  |  |  |  |  |
| --- | --- | --- | --- | --- | --- | --- | --- | --- |
| **Trait, DAS** | **Year** | **Yield** | **Biomass** | **HI** | **CHM 95** | **CHM 110** | **CHM 126** | **CHM 137** |
| **Yield** | **0.64**  *** |  |  |  |  |  |  |  |
| **Biomass** | **0.06**  0.7423 | **0.45**  * |  |  |  |  |  |  |
| **HI** | **0.59**  *** | **0.67**  *** | **-0.31**  0.0817 |  |  |  |  |  |
| **CHM, 95** | **0.23**  0.2037 | **0.23**  0.2080 | **0.25**  0.1655 | **0.01**  0.9712 |  |  |  |  |
| **CHM, 110** | **0.46**  ** | **0.42**  * | **0.32**  0.0768 | **0.15**  0.4193 | **0.84**  *** |  |  |  |
| **CHM, 126** | **0.59**  *** | **0.64**  *** | **0.32**  0.0706 | **0.38**  * | **0.7**  *** | **0.91**  *** |  |  |
| **CHM, 137** | **0.66**  ** | **0.74**  *** | **0.29**  0.1063 | **0.51**  ** | **0.62**  *** | **0.81**  *** | **0.96**  *** |  |
| **Plant height maturity** | **0.44**  * | **0.07**  0.7034 | **-0.15**  0.4035 | **0.21**  0.2413 | **0.11**  0.5631 | **0.06**  0.7577 | **0.08**  0.6467 | **0.19**  0.3051 |

**Supplementary Table 8.** Pearson correlation of year of release (YOR), yield, biomass, CO_2_ assimilation rate (*A*), stomatal conductance (*g_s_*) at flowering, and canopy temperature depression (CTD) from season 2020. This correlation does not include Eston and Indianhead since *A* and *gs* were not collected, df=28.

|  |  |  |  |  | **2020** |  |  |  |  |  |  |  |
| --- | --- | --- | --- | --- | --- | --- | --- | --- | --- | --- | --- | --- |
| **Trait, DAS** | **YOR** | **Yield** | **Biomass** | ***A* flw** | ***g_s_* flw** | ***A pod*** | ***g_s_* pod** | **CTD**  **127** | **CTD**  **138** | **CTD**  **158** | **CTD**  **165** | **CTD**  **173** |
| **Yield** | **0.54**  ** |  |  |  |  |  |  |  |  |  |  |  |
| **Biomass** | **0.25**  0.1798 | **0.46**  * |  |  |  |  |  |  |  |  |  |  |
| ***A* flw, 130-139** | **-0.41**  * | **-0.19**  0.3175 | **-0.01**  0.9504 |  |  |  |  |  |  |  |  |  |
| ***g_s_* flw, 130-139** | **-0.47**  ** | **-0.19**  0.3154 | **-0.05**  0.8103 | **0.86**  *** |  |  |  |  |  |  |  |  |
| ***A* pod, 158-169** | **0.26**  0.1673 | **0.27**  0.1464 | **0.21**  0.2541 | **0.05**  0.7931 | **0**  0.9832 |  |  |  |  |  |  |  |
| ***g_s_* pod, 158-169** | **0.2**  0.3006 | **0.22**  0.2383 | **0.25**  0.1745 | **0.08**  0.6607 | **0.01**  0.9639 | **0.96**  *** |  |  |  |  |  |  |
| **CTD, 127** | **-0.47**  ** | **0.13**  0.4911 | **0.03**  0.8910 | **0.42**  * | **0.53**  ** | **-0.23**  0.2211 | **-0.28**  0.1327 |  |  |  |  |  |
| **CTD, 138** | **0.25**  0.1744 | **0.59**  *** | **0.44**  * | **0.02**  0.9120 | **0.15**  0.4373 | **0.29**  0.1257 | **0.2**  0.2911 | **0.19**  0.3089 |  |  |  |  |
| **CTD, 158** | **-0.33**  0.0781 | **-0.15**  0.4206 | **-0.46**  ** | **0.08**  0.6902 | **0.21**  0.2674 | **0.04**  0.8175 | **0.03**  0.8810 | **0.24**  0.2057 | **-0.1**  0.6056 |  |  |  |
| **CTD, 165** | **-0.35**  0.0589 | **-0.33**  0.0734 | **-0.54**  ** | **0.08**  0.6719 | **0.2**  0.2887 | **-0.09**  0.6282 | **-0.13**  0.4976 | **0.08**  0.6659 | **-0.24**  0.2001 | **0.68**  *** |  |  |
| **CTD, 173** | **0.01**  0.9568 | **-0.16**  0.4047 | **-0.43**  * | **-0.09**  0.6404 | **-0.04**  0.8542 | **-0.02**  0.9229 | **-0.07**  0.7072 | **-0.15**  0.4142 | **-0.23**  0.2266 | **0.63**  *** | **0.63**  *** |  |
| **Days to flowering** | **-0.08**  0.6758 | **-0.1**  0.6004 | **0.19**  0.3092 | **0.13**  0.4810 | **0.08**  0.6678 | **0.09**  0.6298 | **0.1**  0.5886 | **-0.01**  0.9774 | **0**  0.9889 | **-0.33**  0.0791 | **-0.07**  0.6987 | **-0.28**  0.1280 |

**Supplementary Table 9.** Pearson correlation of year of release (YOR), Yield, Biomass, canopy temperature depression (CTD) at 126, 136, 143, 153 and 166 days after sowing (DAS), and days to flowering from season 2021, df=30.

|  |  |  | **2021** |  |  |  |  |  |
| --- | --- | --- | --- | --- | --- | --- | --- | --- |
| **Trait, DAS** | **Year** | **Yield** | **Biomass** | **CTD**  **123** | **CTD**  **136** | **CTD**  **143** | **CTD**  **153** | **CTD**  **166** |
| **Yield** | **0.64**  *** |  |  |  |  |  |  |  |
| **Biomass** | **0.06**  0.7423 | **0.45**  ***** |  |  |  |  |  |  |
| **CTD, 123** | **-0.09**  0.6169 | **0.24**  0.1769 | **0.2**  0.2655 |  |  |  |  |  |
| **CTD, 136** | **0.08**  0.6741 | **0.24**  0.1917 | **0.04**  0.8455 | **0.24**  0.1771 |  |  |  |  |
| **CTD, 143** | **0.35**  ***** | **0.24**  0.1807 | **-0.05**  0.7753 | **0.18**  0.3134 | **0.57**  ******* |  |  |  |
| **CTD, 153** | **0.31**  0.0849 | **0.2**  0.2747 | **-0.17**  0.3408 | **-0.15**  0.4241 | **0.31**  0.0818 | **0.43**  * |  |  |
| **CTD, 166** | **-0.14**  0.4583 | **-0.21**  0.2457 | **-0.04**  0.8088 | **-0.17**  0.3387 | **-0.37**  * | **-0.47**  ** | **0.18**  0.3182 |  |
| **Days to flowering** | **-0.27**  0.1360 | **-0.14**  0.4334 | **-0.03**  0.8513 | **-0.1**  0.6036 | **-0.46**  ** | **-0.5**  ** | **-0.13**  0.4749 | **0.4**  * |
